# High Current Induction for the Effective Bending in Ionic Polymer Metal Composite

**DOI:** 10.3390/membranes15110333

**Published:** 2025-11-03

**Authors:** Hirohisa Tamagawa, Rintaro Fujiwara, Iori Kojima

**Affiliations:** 1 Department of Mechanical Engineering, Faculty of Engineering, Gifu University, 1-1 Yanagido, Gifu 501-1193, Japan; fujiwara.rintaro.e7@s.gifu-u.ac.jp; 2 Nagoya Railroad Co., Ltd., 4-8-26, Meieki, Nakamura-ku, Nagoya-shi 450-8501, Japan; kozima0927@gmail.com

**Keywords:** IPMC, bending, current, electrode, doping, oxygen

## Abstract

Ionic Polymer–Metal Composites (IPMCs) are promising electroactive polymers for artificial muscles, as their bending motion depends on the induced current—greater current leads to greater bending. While conventional IPMCs use cation exchange membranes, this study explores IPMCs containing both immobile positive and negative charges, resembling real muscle tissue. Considering that an IPMC consists of an ion-exchange membrane sandwiched between two thin metal coatings serving as electrodes, we found that (i) improving the contact between the metal coating (electrode) and the ion exchange membrane is an effective way to enhance current induction. Achieving tight electrode membrane contact can drastically increase the induced current by up to four orders of magnitude, and even samples that previously showed no current induction can exhibit measurable current after improvement. (ii) Doping with mobile ions is another well-known method of enhancing IPMC current. However, we found that simply introducing dopants into the IPMC body is not effective; the choice of dopant is crucial. In this work, we identified silver ions as effective dopants for enhancing current induction. Considering that real muscles consume oxygen for activation, we also attempted to supply oxygen to the IPMC surface. We confirmed that (iii) supplying oxygen to the IPMC surface is another effective means of enhancing current induction, which in turn resulted in a significant improvement in IPMC bending performance.

## 1. Introduction

Electrically deformable polymers are promising candidates for artificial muscles, commonly referred to as electroactive polymers (EAPs) [1,2,3,4,5,6,7]. Research on EAPs has been ongoing for several decades, and today, various types are available, including hydrogels, dielectric elastomer actuators (DEAs), and conducting polymers. Since biological muscles are primarily composed of polymers and function through electrical signals transmitted by neurons, EAPs bear a strong resemblance to natural muscles. It is therefore quite reasonable that they are often referred to as artificial muscles.

The origins of EAP research are sometimes traced back to the work of Katchalsky [8,9,10]. In 1949, he demonstrated that collagen filaments could undergo reversible volume changes when immersed in acidic or alkaline solutions—a phenomenon now recognized as a form of mechanochemical response. While not electroactive in the modern sense, this pioneering work laid the foundation for subsequent research into soft polymer-based actuators.

Another major milestone was the discovery of phase transitions in hydrogels by Tanaka [11,12]. This breakthrough led to intensive investigations into the properties of hydrogels, driven not only by scientific curiosity but also by their potential for practical applications. The substantial volume change that hydrogels can undergo has become a key area of focus in EAP research. Although the development of practical polymer-based soft actuators is still ongoing, extensive research into EAPs continues to advance the field. Ionic Polymer Metal Composite (IPMC) is also one of the EAPs. The structure of IPMC is quite simple, and it can be fabricated by coating the top and bottom surfaces of an ion exchange membrane with metal. Despite such a simple structure, IPMC exhibits significant bending even under a quite low voltage. Hence, the IPMC has attracted much attention due to its promising features as a practical soft actuator for more than the past three decades [13,14,15]. A typical IPMC is Nafion-based. Nafion is a cation exchange membrane, which holds immobile negative charges. In fact, the first reported IPMC was made from Nafion [13], and since then a great many scientists have worked on the Nafion-based IPMC especially for the purpose of fabricating a paractical soft actuator and sometimes it is called “artificial muscle” [13,14,15,16,17,18,19]. It exhibits remarkably large bending under electrical stimulation. The electrical stimulation required for bending induction is comparable to the power of a single AAA battery. Broadly accepted bending mechanism of IPMC states that the hydrated mobile cations, which are generated by the dissociation of immobile sulfonic groups contained in the Nafion of the IPMC, are attracted toward cathode when the exrernal voltage is exerted to the IPMC. Therefore, the cathode side of the IPMC swells while its anode side contracts due to the loss of hydrated mobile cations. Consequently, the Nafion-based IPMC bends in the anode direction [15,19]. It is naturally speculated that any ion exchange membrane can serve as the matrix for an IPMC, since it can generate the mobile hydrated ions in the wet state. In fact, not only Nafion but also other types of ion-exchange membranes can be used as the matrix for IPMC [16,17,18]. Tamagawa et al. also studied several different types of IPMCs, including those made with positively charged ion exchange membranes, negatively charged ion exchange membranes and a bipolar ion exchange membrane [20]. Here we would like to touch upom their studies about the four kinds of IPMCs as follows: The first is the Selemion CMV-based IPMC. Selemion CMV is a cation exchange membrane manufactured by Asahi Glass Co., Ltd. (Japan). The membrane was coated with silver to produce the IPMC. In addition to Selemion CMV, Tamagawa et al. fabricated IPMCs using Selemion CMVN, Selemion AMV, and Selemion AMVN by coating their surfaces with silver. Selemion CMVN is also a cation exchange membrane, whereas Selemion AMV and Selemion AMVN are anion exchange membranes. All of these membranes are also produced by Asahi Glass Co., Ltd. (Japan). All four types of IPMCs exhibited bending in response to external electrical stimulation. However, the degree of bending was not always sufficient, and control of the bending through electrical stimulation was found to be far from practically effective. Achieving a truly practical IPMC remains a distant goal. To make progress toward this, we further investigated the characteristics of IPMCs.

To achieve practical IPMC performance, one of the essential characteristics is effective bending. Previous studies suggest that high current induction can lead to effective bending [21,22,23]. Therefore, it is natural to consider that we should find a way to enhance current induction to achieve effective bending. A well-known method for achieving this is doping, which involves introducing mobile ions into the IPMC body [24,25], and acually doped IPMCs exhibit effective bending with enhanced current [21,22].

Since EAPs are often referred to as artificial muscles, it is important to emphasize their similarities with biological muscles. Biological muscles are essentially composed of polymers (proteins) that contain both positively and negatively charged immobile groups, and, as previously mentioned, their actions are governed by electrical signals transmitted through neurons. Given this analogy, we chose to investigate the bipolar IPMC in greater detail, as their polymer matrix also contains both positively and negatively charged immobile groups. The bipolar IPMC we investigate in this work is a silver-coated bipolar ion exchange membrane. Previously, we already studied the characteristics the bipolar IPMC and observed it exhibited bending by the voltage application like the commonly invesigated Nafion-based IPMC [20]. But it is still unknown what causes the bending exactly. Our previous work suggests that various IPMCs of silver-coated ion exchage membranes (cation echange membrane type, anion exhage membrane type and bipolar membrane type) were always accompanied by the silver redox reaction when they exhibit effective bending, and we also found that the effective current induction occurs when the silver redox reaction was induced [22,23]. Therefore, it is quite natural to be motivated to investigate the relationship between the current induction and bending of IPMC.

Our IPMC study has been guided by the physiological characteristics of muscles, with the aim of contributing to the development of practical and functional IPMCs. In addition, we focus on the essential role of oxygen in sustaining life, particularly its well-established importance in muscle activity, which inherently requires oxygen consumption [26,27,28,29]. Based on this, we hypothesized that oxygen utilization could enhance the bending performance of artificial muscles, such as IPMCs—though this may initially appear speculative. Nevertheless, we present scientific observations and a conceptual framework that link oxygen to the activation of both biological and artificial muscles, as outlined below.

**Artificial muscle**: IPMC represents a class of artificial muscles. One of the authors of this paper, Tamagawa, along with his former colleagues, fabricated an IPMC using a silver-coated Selemion CMV, where Selemion CMV is a cation exchange membrane manufactured by Asahi Glass Co., Ltd. (Tokyo, Japan). This IPMC exhibits significant bending in response even to quite low-voltage stimulation. During actuation, the silver layer on the membrane surface undergoes a redox reaction, represented by the equation: 4Ag + O_2_ ⇌ 2Ag_2_O. Concurrently, a notable increase in current is observed [22,23]. These findings suggest that effective bending is strongly linked to the silver redox reaction and the associated increase in current flow, indicating that oxygen plays a critical role in the actuation behavior of IPMCs.
**Biological muscle**: The Murburn concept is a novel biological hypothesis proposed by K. M. Manoj in the 2010s [30,31,32,33]. Traditionally, reactive oxygen species (ROS) have been considered harmful byproducts of metabolism. However, according to the Murburn concept, ROS functions as essential mediators in electron transfer processes. Therefore, it is reasonable to speculate that the activation of biological muscles involves oxygen and ROS more integrally than ordinarily believed.

Therefore, we attempt to find the conditions under which the high current can be induced by focusing on the treatment of doping of IPMC and the utlization of oxygen supply on the IPMC body.

## 2. Materials and Methods

### 2.1. Specimen Preparation

*Fabrication of IPMC*: Neosepta is an ion exchange membrane manufactured by ASTOM Corporation in Tokyo, Japan (https://www.astom-corp.jp/en/). Its characteristics is featured by that it contains both immobile negative charge and positive charges [34,35] while Nafion contains only the immobile negative charges. Neosepta is a bipolar ion exchange membrane. Electrical characteritics of bipolar materials may play important role for achieving the practical IPMC actuator because the biological muscle consits of a lot of polymers (proteins) which bear a lot of immobile cations and immobile anions. So, Neosepta-based IPMC could have quite similar charatcteritics to the biological muscle, that is, the Neosepta-based IPMC could serve as a quite effective artificial muscle.

**Figure 1:**
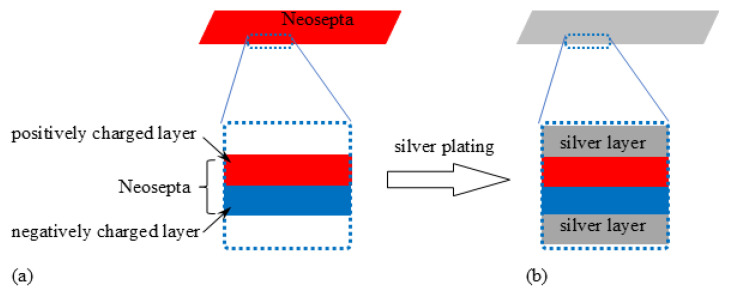
(**a**) Structure of bipolar membrane, Neosepta, denoted as BP. (**b**) Structure of Neosepta-based IPMC, BP-IPMC.

Neosepta comprises two layers: a positively charged layer and a negatively charged layer, as illustrated in Figure 1a. The surface is coated with silver using the well-known silver mirror reaction [21]. The resulting IPMC structure is shown in Figure 1b. This IPMC is hereafter referred to as BP-IPMC (abbreviation of “BiPolar ion exchange membrane-based Ionic Polymer Metal Composite”). The dimensions of BP-IPMC are 20 mm length × 2 mm width. The IPMC ingredient Neosepta which had not undergone silver mirror reaction is to be called BP from now on.

*Fabrication of doped BP*: Pieces of BP were immersed in 1 M electrolyte solutions for doping. The 1 M electrolyte solutions we prepared were CaCl_2_, MgCl_2_, and AgNO_3_. The BPs doped with CaCl_2_, MgCl_2_, and AgNO_3_ are referred to as BP_*C*_, BP_*M*_, and BP_*A*_, respectively. The BP contaning the deionized water only is denoted as BP_*w*_. Please note that these are not IPMCs, but rather pieces of ion-doped BP from Neosepta.

### 2.2. Experimental Methods

#### 2.2.1. Bending Curvature Measurement

**Figure 2:**
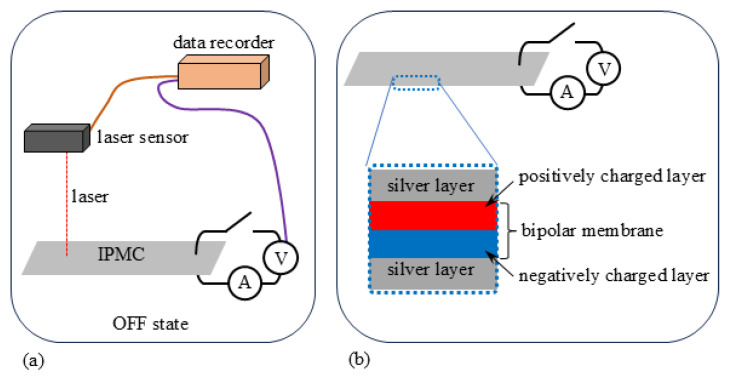
(**a**) Experimental setup for measuring the bending response of the IPMC under an applied voltage. A data recorder (LR8431, HIOKI E.E. Corporation, Nagano, Japan), a laser sensor (IL-030, Keyence, Osaka, Japan), and devices for voltage and current control and measurement, including a potentiostat/galvanostat (HA-151B, Meiden Hokuto Corporation, Hyogo, Japan) and a signal generator (PSG9080, Hangzhou Junce Instruments Co., Ltd., Hangzhou, China), were used. Further detail of the experiemntal setup is given in Appendix A. (**b**) Side view of the clamped IPMC, showing which surface is facing upward or downward.

For measuring the bending characteritics of BP-IPMC, the experimental setup illustrated in Figure 2 was used (see Appendix A as well). BP-IPMC was horizontally clamped between a pair of electrodes (see Figure 2a) so that the positively charged layer of faces up and the negatively charged layer faced down as illustrated in Figure 2b.

**Figure 3:**
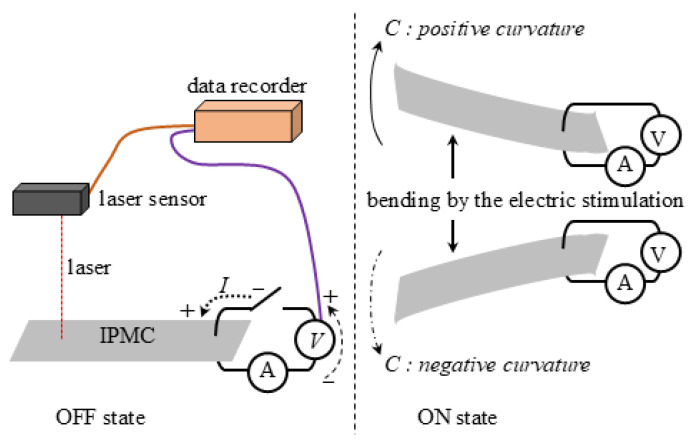
Definitions of the positive direction of voltage, current, and the curvature of IPMC.

**Figure 4:**
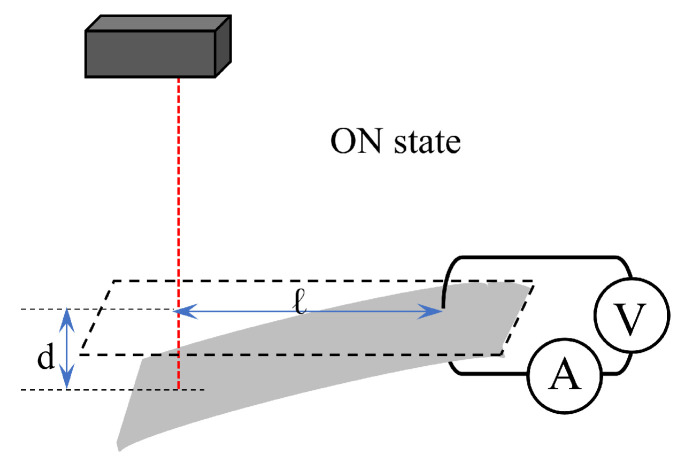
The IPMC is in a downward-bending state. *ℓ* represents the distance from the point where the IPMC is clamped between a pair of electrodes to the laser, and *d* represents the vertical displacement caused by the bending (*d* is negative in this illustration).

It needs to define the poistive and negative of voltage (*V*), current (*I*) and curvature (*C*). Figure 3 illustrates the positive direction of voltage and current, as well as the positive of IPMC curvature: a counter-clockwise direction for voltage and current is defined as positive, and upward bending curvature is also defined as positive. Figure 4 illustrates the definitions of the distance (*ℓ*) from the point where the IPMC is clamped between a pair of electrodes to the laser, and the vertical displacement (*d*) of the IPMC. In Figure 4, a negative value of *d* is shown as an exampe and it indicates downward displacement, meaning that upward displacement is defined as positive. The curvture is computed by plugging the experimental data of *d* and *ℓ* into the following equation.

(1) $$\begin{array}{c} C=\frac{2d}{d^{2}+\ell^{2}} \end{array}$$

#### 2.2.2. Current Measurement

**Figure 5:**
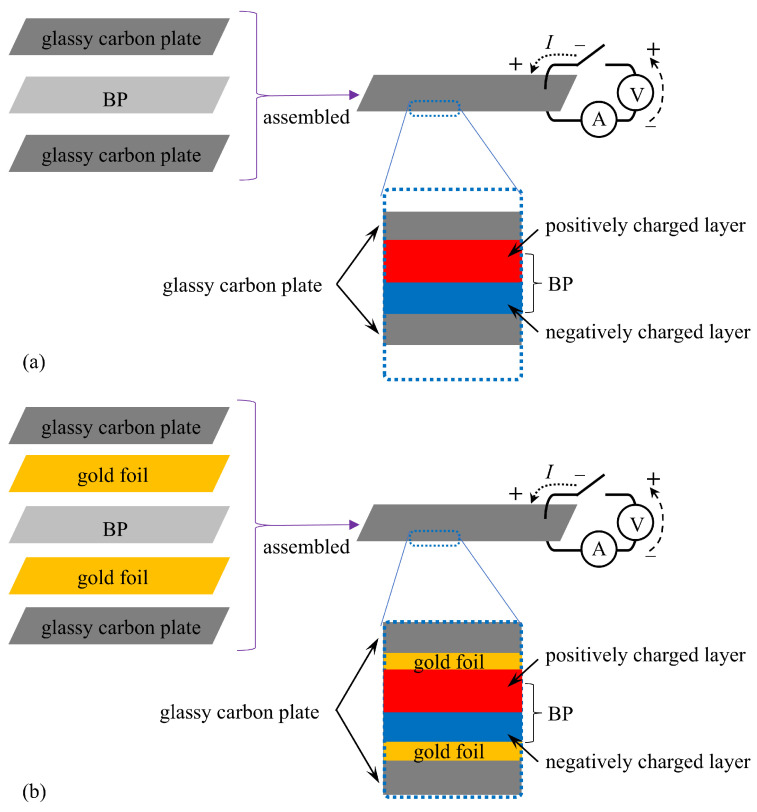
A setup for measuring the current through doped BP, along with a side view of the specimen. (**a**) A BP specimen is sandwiched between a pair of glassy carbon plates. (**b**) A BP specimen is sandwiched between a pair of glassy carbon–gold foil plates. The definitions of the positive and negative directions for voltage and current are also indicated.

*Current measurement for BP_i_*: The current induced in the BP_*i* (*i*=*C*, *M*, *A*, *w*)_ (excluding the BP-IPMC) was measured by sandwiching the specimen between two glassy carbon plates, as shown in Figure 5a. A voltage was applied, and the resulting current was recorded over time. This procedure was repeated for all other specimens. Since all the BP_*i*_ were fully sandwiched between the glassy carbon plates, no bending deformation occurred during the measurements. The same current measurement was conducted using the slightly different electrode as illustrated in Figure 5b. Two pieces of gold foil were used. The reason for using these gold foils will be explained with reference to Figure 14.

**Figure 6:**
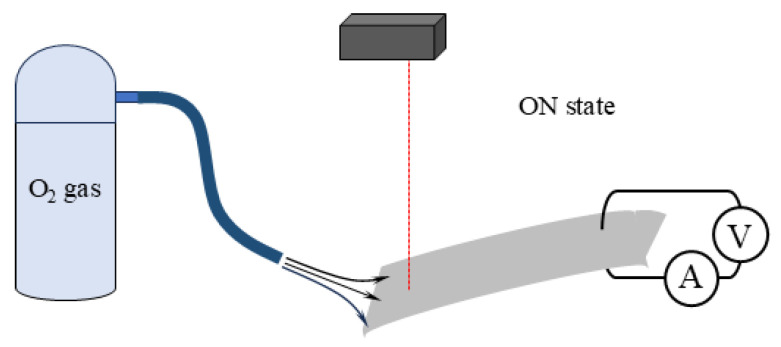
BP-IPMC bending tests were conducted with oxygen gas being blown onto the BP-IPMC using this setup.

*Current measurement for BP-IPMC under the oxygen*: We conducted another current measurement under the oxygen supply to the BP-IPMC (excluding BP_*i*_). The high current could be induced under the oxygens supply as described at the end of the Section 1. The experimental setup is illustrated in Figure 6. Since no glassy carbon plates (and no gold foils) were used as the electrodes, BP-IPMC exhibited bending during this current measdurement.

### 2.3. Environmental Conditions in the Lab

Here, we would like to refer to the environmental conditions in the laboratory. The laboratory temperature was maintained at 298 K throughout the year, and the experiments described in this paper were conducted when the absolute humidity was approximately 6 g/m^3^.

## 3. Results and Discussions

### 3.1. Characteritics of BP-IPMC

**Figure 7:**
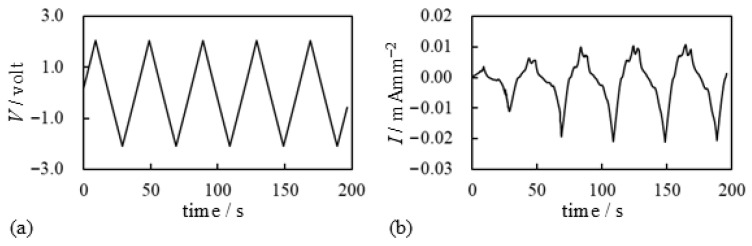
Electrical characteristics of BP-IPMC. (**a**) Voltage vs. time. (**b**) Current vs. time.

First, we conducted bending tests of the BP-IPMC using the setup illustrated in Figure 2. Please note that the oxygen supply illustrated in Figure 6 was not used in this case, as the setup shown in Figure 2 was employed. The definitions of the positive and negative directions of voltage, current, and curvature are shown in Figure 3. Figure 7 presents the voltage applied to the BP-IPMC and the resulting induced current. Although the applied voltage profile is symmetric about *V* = 0 V, the current response is asymmetric with respect to *I* = 0 mA mm^−2^. The magnitude of the negative current is significantly greater than that of the positive current.

**Figure 8:**
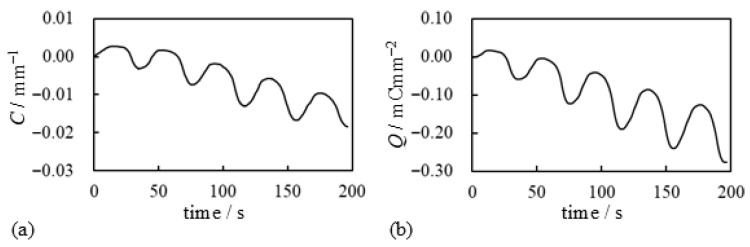
Electrical characteristics of BP-IPMC obtained when the experiment for obtaining Figure 7 was conducted (**a**) Curvature vs. time (**b**). Charge vs. time.

**Figure 9:**
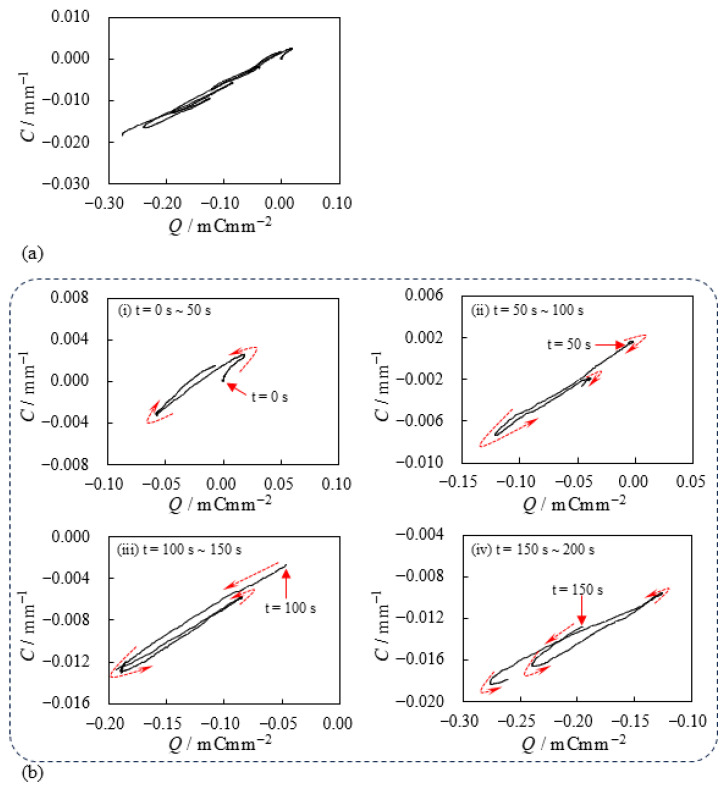
Curvature vs. Charge of BP-IPMC. The diagram (**a**) was obtained by arranging the data in Figure 8. Panel (**a**) is segmented into 50-s intervals and displayed at higher magnification in (**b**), with dashed arrows indicating the direction of time.

As described in Section 1, the current can be a governing factor in the bending behavior of IPMCs. Therefore, an asymmetric current profile may result in an asymmetric bending curvature about *C* = 0 mm^−1^. As expected, such a curvature profile was obtained, as shown in Figure 8a. Figure 8b shows the charge Q(t) as a function of time *t*, where Q(t) was calculated by integrating the experimentally measured current I(t), shown in Figure 7b, using Equation (2). The data from Figure 8 were then replotted to yield the relationship between curvature *C* and charge *Q*, as shown in Figure 9. Figure 9a shows that the curvature is proportional to the total charge applied to the BP-IPMC, and the slope of this relationship is positive. This proportional relationship with a positive slope is a typical characteristic of IPMCs composed of a cation exchange membrane coated with silver. In contrast, a negative slope in the *C*–*Q* relationship has been observed for IPMCs composed of an anion exchange membrane coated with silver [20]. Therefore, the bending characteristics of the BP-IPMC are largely governed by the properties of the negative immobile charge layer, which plays a role as the cation exchange membrane, in the Neosepta part of the BP-IPMC.

(2) $$\begin{array}{c} Q\left(t\right)=\int_{0}^{t}I\left(\tau \right)d\tau \end{array}$$

### 3.2. Current of BPs

#### 3.2.1. Doping

Our research group has recognized that IPMC bending is largely governed by the induced current (or total charge), as reported in previous studies [22,36], although IPMC bending tests are typically conducted under voltage control [1,2,13,14,15,17,18,20,24]. As described in Section 1, current induction can be enhanced by doping the IPMC [21,22,23,24,25]. Therefore, we investigated the current characteristics of doped BP_*i*_ (not BP-IPMC) by following the procedure described in Section 2.2.2.

**Figure 10:**
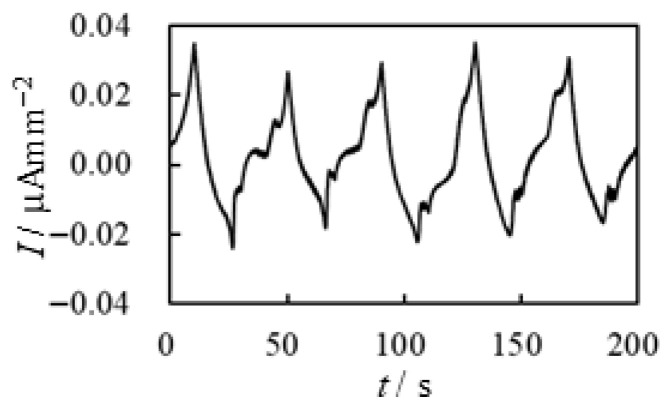
Current vs. Time about the BP_*A*_ under the voltage shown in Figure 7a.

A piece of BP_*i*_ was sandwiched between a pair of glassy carbon electrodes, as illustrated in Figure 5a. The voltage profile applied to the BP_*i*_ was the same as that shown in Figure 7a. None of BP_*C*_, BP_*M*_, or BP_*w*_ exhibited measurable current; virtually no current was observed. However, BP_*A*_ exhibited a faint current, as shown in Figure 10, where the vertical axis represents current per unit area of the BP_*A*_. Nevertheless, the current level remained very low (see the units on the vertical axis, μA mm^−2^). Interestingly, the positive current of BP_*A*_ appears to be slightly greater than the negative current, as seen in Figure 10, which contrasts with the current profile of BP-IPMC shown in Figure 7b. We will discuss this observation later.

**Figure 11:**
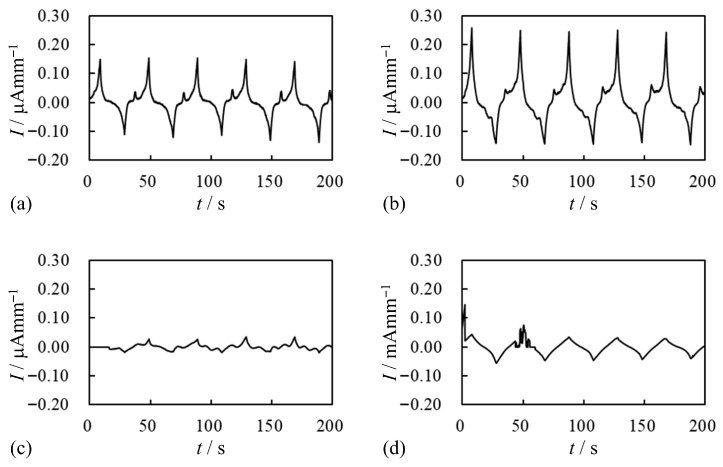
Current vs. Time about BP_*i*_ under the voltage shown in Figure 7a. (**a**) BP*_w_*. (**b**) BP_M_. (**c**) BP__C__. (**d**) BP*_A_*. Unit of the current of (**a**–**c**) is “μA mm^−2^” while that of (**d**) is “mA mm^−2^”.

Next, the same current measurement was performed on the same BP_*i*_ using a different experimental setup, as illustrated in Figure 5b. The difference between the setups in Figure 5a,b lies in the use of gold foils. The results are shown in Figure 11. Compared to the current observed with the setup in Figure 5a, the current measured using the setup in Figure 5b was significantly enhanced. Among the four current profiles in Figure 11, the current of BP_*A*_ is particularly high compared to the others.

The current of BP_*C*_ shown in Figure 11c appears quite low, even in comparison to the currents of BP_*w*_ and BP_*M*_ in Figure 11a and Figure 11b, respectively. However, although the currents of BP_*w*_ and BP_*M*_ are higher than that of BP_*C*_, they are still relatively low in absolute terms. Therefore, we do not consider the differences in current magnitudes among BP_*w*_, BP_*M*_, and BP_*C*_ to be significant.

**Figure 12:**
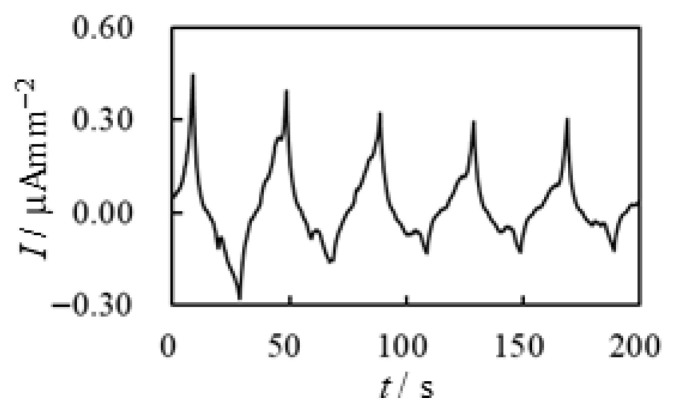
Current vs. Time about the BP*_C_* under the same condition employed for obtaining Figure 11c.

In fact, repeated measurements revealed that the magnitude of the induced current varies from specimen to specimen, even among samples of the same type. Nevertheless, the overall trend of current levels remains relatively consistent. To illustrate this point, we present the “Current vs. Time” profile of another BP_*C*_ specimen in Figure 12. The current in this diagram is higher than that shown in Figure 11c, although both represent BP_*C*_ samples.

We intentionally include both profiles—one with a relatively low current (Figure 11c) and one with a relatively high current (Figure 12)—to emphasize that the low current in Figure 11c does not imply that BP_*C*_ inherently exhibits lower currents than BP_*w*_ or BP_*M*_. As previously mentioned to the effect that *“the magnitude of the induced current varies from specimen to specimen, although the overall order of the observed current levels remains relatively consistent”*, even among samples of the same type, such as the BP_*C*_s used to obtain Figure 11c and Figure 12. The same is true for BP_*w*_ and BP_*M*_. On the other hand, the current of BP_*A*_ shown in Figure 11d is clearly enhanced when using the setup shown in Figure 5b compared to the current of BP_*A*_ shown in Figure 10 which was obtained using the setup illustrated in Figure 5a. The current improved by about four orders of magnitude.

**Figure 13:**
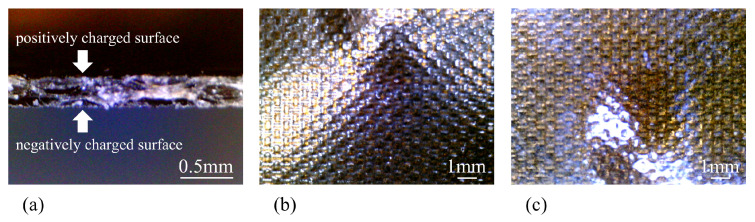
(**a**) cross section of Neosepta. (**b**) positively charged side of Neosepta surface. (**c**) negatively charged side of Neosepta surface.

**Figure 14:**
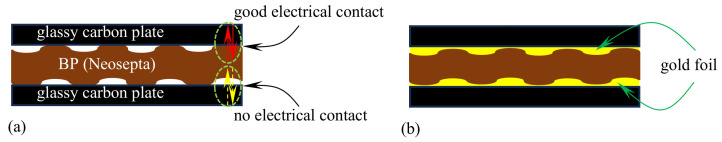
Side view of Neosepta (BP)-electrode interface (**a**) when the setup illustrated in Figure 5a in use. (**b**) when the setup illustrated in Figure 5b in use.

Regarding Figure 10, we previously stated that we would discuss this current profile in more detail, and we do so here. As mentioned earlier for BP_*w*_, BP_*M*_, and BP_*C*_ in Figure 11, their current magnitudes should be considered similarly low, even though the currents of BP_*w*_ and BP_*M*_ appear higher than that of BP_*C*_ at first glance. In the same way, the current of BP_*A*_ shown in Figure 10 remains within the low-current range, despite appearing more prominent. As demonstrated by the experimental results, the use of the setup illustrated in Figure 5b enhances current generation compared to the setup in Figure 5a, indicating that the gold foil electrode clearly promotes current induction. But why is the gold foil so important for effective current induction? We believe that the key factor is the quality of contact between the BP_*i*_ surface and the electrode. Figure 13a shows a cross-sectional view of Neosepta, the base material used in BP_*i*_ and BP-IPMC in this study. A small peice of Neosepta was cut with the sharp knife and its crosssectional image was taken using the optical microscope. Its surfaces which are shown in Figure 13b,c are highly irregular, with pronounced concave and convex features. In contrast, the glassy carbon plate used in the setup in Figure 5a is extremely flat and mechanically rigid, making it unlikely that the BP_*i*_ surface can achieve good electrical contact with it. This poor contact condition is illustrated in Figure 14a. In contrast, the gold foil used in Figure 5b is very soft and flexible due to its material properties and fine thickness (0.1–0.2 μm). It can deform and conform to the irregular BP_*i*_ surface, as illustrated in Figure 14b, thereby creating effective electrical contact between the BP surface and the glassy carbon electrode via the gold foil. Therefore, choosing an appropriate electrode material is a fundamentally important factor in enhancing current induction, and consequently, in improving IPMC bending performance.

Here, we discuss the doping treatment. We already discussed that the current of BP_*w*_, BP_*C*_ and BP_*M*_ shown in Figure 11 are same one another. In fact, BP_*w*_ is not doped specimen, but the current of doped BPs of BP_*C*_ and BP_*M*_ are the same level of the current of BP_*w*_. It is interepreted that the doping is ineffective for the current induction. However, the current of BP_*A*_ is extremely greater than that of BP_*w*_, BP_*C*_ and BP*_M_*. Therefore, it needs to choose appropriate ions for the realization of meningful doping. For our study, the effective dopant was found to be AgNO_3_.

#### 3.2.2. Oxygen Supply

The current through the BP-IPMC during bending under an applied voltage was measured in the setup shown in Figure 6, with oxygen gas being blown onto the IPMC surface. For comparison, the same measurement was performed using nitrogen gas instead of oxygen. According to the experimental results of the BP-IPMC bending test shown in Figure 7, the magnitude of the negative current exceeds that of the positive current. Therefore, we chose a constant negative voltage of −2 V to be applied to the BP-IPMC while oxygen was blown onto its surface, as illustrated in Figure 6.

**Figure 15:**
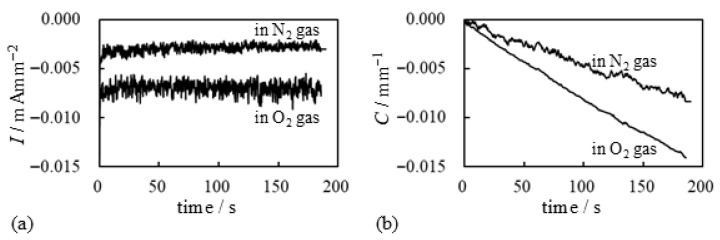
Electrical characteritics of BP-IPMC. (**a**) bending curvature vs. time and (**b**) current vs. time of BP-IPMC in O_2_ and N_2_ atmosphere under a constant −2 V applied voltage.

Figure 15 presents the current and bending curvature response of the BP-IPMC under a constant applied −2 V voltage with external oxygen supply. The same figure also displays the results obtained when nitrogen was used in place of oxygen. As expected, Figure 15a shows that the current magnitude is larger with O_2_ than with N_2_. As previously discussed, this higher current likely contributes to increased bending of the IPMC. Indeed, Figure 15b indicates that the bending curvature is larger when O_2_ gas is used. Hence, the current induction is meaningful for the induction of effective IPMC bending. One might initially assume that the difference in current magnitude between the two diagrams in Figure 15a is not significant, as the discrepancy appears to be only a factor of two. In fact, in Section 3.2.1, we regarded the magnitude of the BP_*C*_ current shown in Figure 11c as being at essentially the same level as that in Figure 12, even though the actual difference in current magnitude was approximately a factor of ten. However, it is important to note that the vertical axis in Figure 15a is labeled in “mA mm^−2^”, whereas those in Figure 11c and Figure 12 are labeled in “μA mm^−2^”. Therefore, the current in Figure 15a cannot be considered negligibly small. On the contrary, it is sufficiently high to be meaningful, and we have experimentally confirmed that the current profiles shown in Figure 15a are quantitatively reproducible.

**Figure 16:**
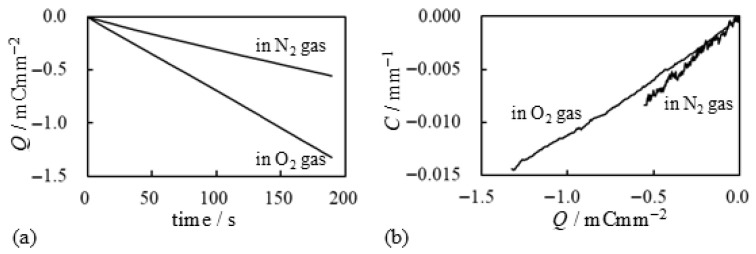
Electrical characteritics of BP-IPMC. (**a**) charge vs. time and (**b**) curvature vs. charge. The diagram (**a**) was obtained by numerically calculating Equation (2) using the experimental data shown in Figure 15a. The diagram (**b**) was obtained arranging the data shown in Figure 15b and Figure 16a.

We conducted a further investigation based on the experimental results. Using the current data shown in Figure 15a, we calculated the time-dependent charge Q(t) using Equation (2), and the results are presented in Figure 16. As illustrated in Figure 16a, the total induced charge in the BP-IPMC is larger when oxygen is supplied. However, Figure 16b indicates that the bending efficiency per unit charge is a bit higher when nitrogen is used. Thus, while oxygen facilitates a greater overall induction, a portion of the charge with oxygen appears to be largely diverted to processes other than bending actuation. Nevertheless, the total charge induced under the oxygen supply is quite large. Hence, the resulting bending curvature becomes greater than that observed when the nitrogen used as shown in Figure 15b.

A related experiment was performed using the setup shown in Figure 6, where a constant –2 V was applied to the BP-IPMC for six minutes. N_2_ and O_2_ gases were alternately introduced according to the timeline shown below (“Timeline of *GAS EXPOSURE EXPERIMENT* under –2 V application”), with t=0 s marking the start of voltage application.

Timeline of *GAS EXPOSURE EXPERIMENT* under –2 V voltage application

- *t* = 0 ∼ 60 s        BP-IPMC is exposed to the N_2_ supply
- *t* = 60 ∼ 120 s       BP-IPMC is in the air without the supply of O_2_ or N_2_.
- *t* = 120 ∼ 180 s     BP-IPMC is exposed to the O_2_ supply
- *t* = 180 ∼ 240 s     BP-IPMC is exposed to the N_2_ supply
- *t* = 240 ∼ 300 s     BP-IPMC is in the air without the supply of O_2_ or N_2_.
- *t* = 300 ∼ 360 s     BP-IPMC is exposed to the O_2_ supply

**Figure 17:**
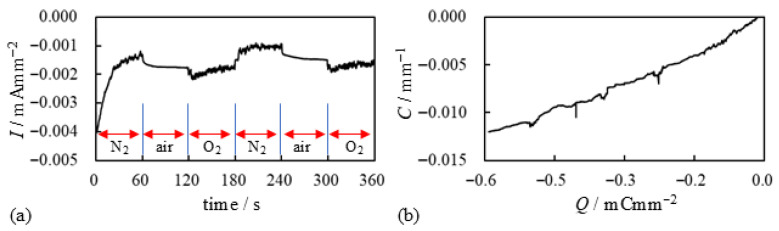
Electrical characteritics of BP-IPMC surrounded by various gases. (**a**) current vs. time (**b**) curvature vs. charge. Conditon of the gas use is described in “Timeline of *GAS EXPOSURE EXPERIMENT* under –2 V voltage application” right before this figure.

We explain the current data shown in Figure 17a. Upon applying the voltage at t=0 s with an N_2_ supply, the current began to decay. At t=60 s, the N_2_ supply was stopped, and the current magnitude increased abruptly—likely due to the presence of oxygen in the ambient air. At t=120 s, a substantial amount of O_2_ was forcibly supplied to the BP-IPMC, resulting in a further sharp increase in current. However, when the O_2_ supply was stopped and a substantial amount of N_2_ was introduced at t=180 s, the current dropped abruptly. This is likely due to the suppression of effective current induction in the absence of O_2_. The subsequent current profile after t=180 s can be interpreted in the same manner. These results indicate that oxygen is fundamentally important for effective current induction. Nevertheless, the relationship between curvature and charge shown in Figure 17b suggests that the bending efficiency per unit charge remains essentially unchanged, even with O_2_ supply. This observation aligns with our earlier comment on Figure 16 to the effect that *a portion of the induced charge in the presence of oxygen appears to be diverted to processes other than bending actuation*. That said, we emphasize once again that *the total charge induced under oxygen supply is significantly higher. Consequently, the resulting bending curvature is greater than that observed under limited or no oxygen supply, as shown in Figure 15b*.

We would like to clarify an important point regarding the current data shown in Figure 17a, which may appear surprisingly different from that in Figure 7b. Namely, the magnitude of the current in Figure 17a is significantly lower than that shown in Figure 7b, despite the voltage employed for obtaning Figure 17a was same or greater than that for obtaining Figure 7b in magnitude. This kind of electrical characteritics difference is a well-known phenomenon in IPMC research, as IPMC characteristics are highly sensitive to environmental humidity [37]. Despite our efforts to maintain the environmental conditions described in Section 2.3, achieving perfectly stable conditions is sometimes difficult. Therefore, somewhat unusual electrical characteristics are occasionally observed in the experiments. Although we have access to an environmental chamber for controlling temperature and humidity, we avoid using it whenever possible due to instability and inconsistency in the artificially controlled environment. Therefore, differences in current magnitude—sometimes varying by a factor of approximately ten—are not unusual. When comparative experimental data are needed, we conduct experiments under similar environmental conditions, including weather, particularly temperature and humidity. Accordingly, we avoid comparing data collected in different seasons, such as summer and winter, unless necessary. It is important to note that the data shown in Figure 17 were obtained under weather conditions and during the same season as those in Figure 11. However, environmental conditions such as temperature and humidity are not relevant for the experiments producing the data in Figure 11, since the BP_*i*_ specimens were completely covered by the electrodes and isolated from environmental influences.

### 3.3. Doping and the Use of Oxygen for Effective Bending

A tight interface between the electrode and the IPMC surface, the use of appropriately selected ions as dopants, and the presence of oxygen can significantly enhance the current in IPMCs. Such enhancement, in turn, can lead to more effective bending performance. Taking these factors into account, we designed an efficiently deformable IPMC. Therefore, we propose that the following points, (i) to (iii), are essential.

*The essential points for achieving the efficiently deformable IPMC*

(i) Electrical tight contact between the surace of Neosepta and the electrode
(ii) Doping with AgNO_3_
(iii) Oxygen supply

BP-IPMC is fabricated by coating the surfaces of Neocepta with silver using the well-known silver mirror reaction, as described in Section 2.1. The silver mirror reaction can tightly coat even complex material surfaces with silver. Therefore, the interface between the silver layer and the Neosepta surface satisfies essential point (i). The fabrication of BP-IPMC involves the silver mirror reaction process, which inevitably impregnates the BP-IPMC with AgNO_3_. Hence, BP-IPMC automatically satisfies essential point (ii).

**Figure 18:**
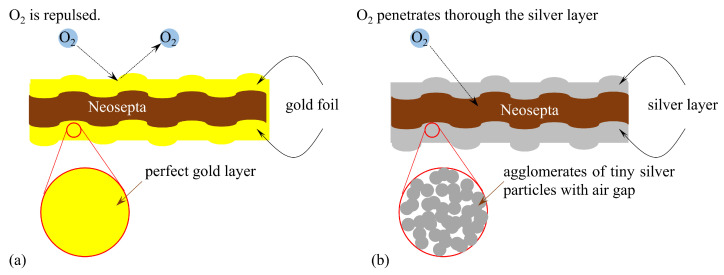
Structure of metal layers of BP-IPMC. (**a**) O_2_ is blocked by the perfectly structured (without defects) gold foil. (**b**) O_2_ penetrates through the silver layer with a lot of voids of air.

**Figure 19:**
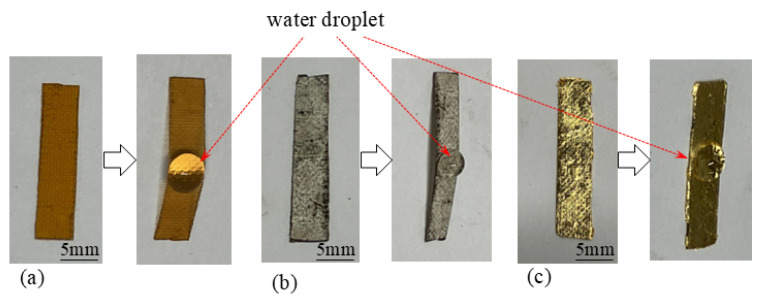
Deformation (and no deformation) of Neosepta due to the water absorpion. (**a**) Non-treated Neosepta. (**b**) Silver-coated Neosepta where the silver coating was formd by the silver mirror reaction. (**c**) Gold foil-coated Neosepta.

If the surface of an IPMC is coated with a metal foil, the foil inevitably prevents external gases from entering the IPMC body. For example, when the IPMC is coated with a gold foil, the gold foil effectively blocks gas penetration into the interior of the IPMC. (see Figure 18a). On the other hand, the silver layer of BP-IPMC is formed merely by piling up silver particles on the surface, hence, there are gaps among silver particles. Therefore, external gases such as oxygen can penetrate through the gaps among the silver particles and reach the Neosepta layer (see Figure 18b). In order to verify this speculation, we conducted the following simple tests. Three small pieces of Neosepta were prepared: one untreated Neosepta (Figure 19a); the second, a silver-coated Neosepta, in which the silver layer was formed by the silver mirror reaction (Figure 19b); and the third, a Neosepta covered with a gold foil (Figure 19c). A small droplet of water was placed on each specimen, and they were left to stand for several minutes. The untreated Neosepta deformed due to water absorption, as shown in Figure 19a. Such deformation induced by water absorption is a well-known phenomenon in ion exchange membranes [38,39]. The silver-coated Neosepta also deformed, as clearly seen in Figure 19b, suggesting that water permeated through the silver layer and reached the Neosepta membrane, resulting in deformation. In contrast, the gold foil–coated Neosepta did not deform even though a water droplet remained on its surface (Figure 19c), indicating that water did not permeate through the gold foil layer and thus could not reach the Neosepta membrane. Thus, we can extend this observation as that the BP-IPMC inevitably benefits from an external oxygen supply for its bending owing to the permeation of oxygen through the voids of silver layers of BP-IPMC and reaches the Nesepta layer. Accordingly, essential point (iii) is effectively fulfilled in the case of BP-IPMC bending.

**Figure 20:**
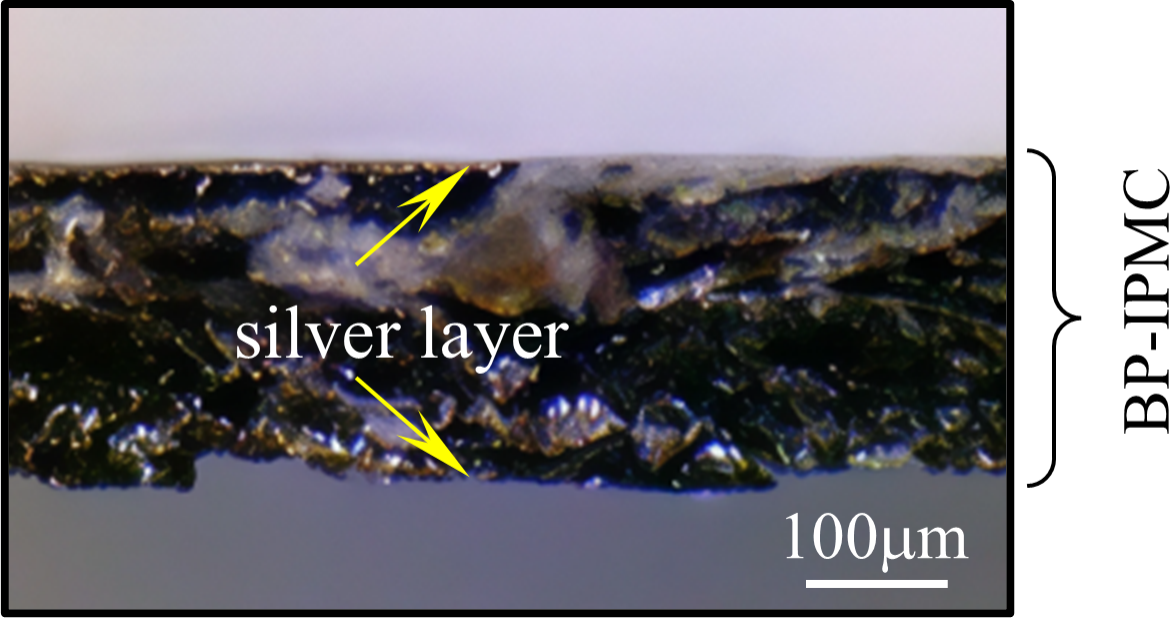
Cross sectional view of BP-IPMC. Thickenss of surface silver layer is ∼30 μm measured using the micrometer.

Next, we examine whether BP-IPMC practically satisfies the essential points (i) to (iii) as follows. Essential point (i) is satisfied in BP-IPMC, as clearly observed in the cross-sectional images shown in Figure 20. Figure 17 demonstrates that essential point (iii), which can be interpreted as the enhancement of BP-IPMC bending due to oxygen supply, is also fulfilled.

**Figure 21:**
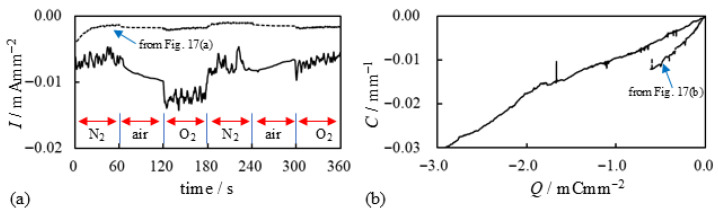
(**a**) Current vs. time of the ADBP-IPMC in the relatively well-dried state and (**b**) Curvature vs. toal charge imposed on it.

Then, how can we practically validate essential point (ii)? BP-IPMC is essentially an IPMC doped with AgNO_3_. To investigate further, we additionally doped BP-IPMC by immersing it in a 1 M AgNO_3_ solution for several hours. This additionally doped BP-IPMC was then left to dry in air for several days. The resulting specimen is hereafter referred to as ADBP-IPMC (Additionally Doped BP-IPMC). ADBP-IPMC underwent the same *Gas Exposure Experiment* as performed to obtain the results shown in Figure 17. The corresponding result is presented in Figure 21a, along with the data from Figure 17a for the BP-IPMC which was not additionally doped. Compared with the BP-IPMC, the magnitude of the induced current in ADBP-IPMC is clearly higher, as seen in Figure 21a. Furthermore, as expected, the final bending curvature of ADBP-IPMC shown in Figure 21b is significantly larger than that of BP-IPMC (also shown in Figure 21b). This suggests that doping IPMC with AgNO_3_ is quite effective for inducing higher current. However, the influence of N_2_, air, and O_2_ on current induction, as shown in Figure 21a, becomes indistinguishable compared to the current of BP-IPMC in Figure 17a, especially after t=180 s. We should also comment on Figure 21b: despite the significant improvement in the final bending curvature of ADBP-IPMC, the efficiency of induced current on bending enhancement appears to worsen with doping. This phenomenon is consistent with the earlier comment on Figure 17b—*a portion of the charge in the presence of oxygen appears to be largely diverted to processes other than bending actuation*.

**Figure 22:**
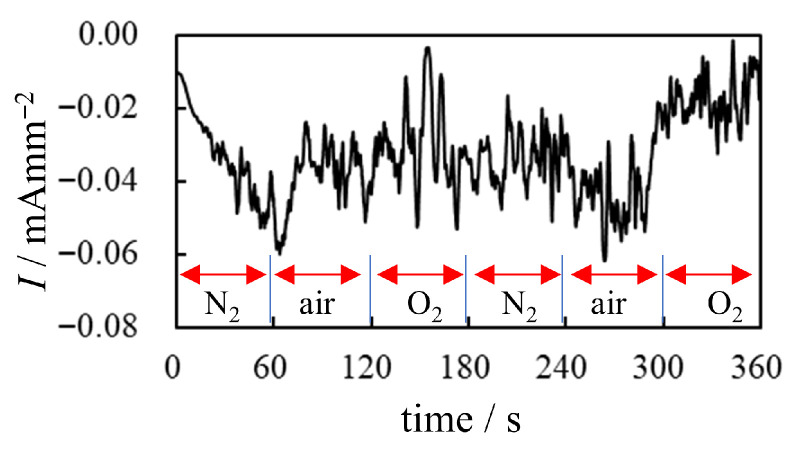
Current vs. time of ADBP-IPMC in the highly wet state.

For comparison, we conducted the same experiment using the ADBP-IPMC, but it was in the wet state; that is, the specimen had not undergone the drying process. In this case, the experiment did not yield reproducible results. The current profiles varied significantly from specimen to specimen. One such result is shown in Figure 22, which appears to contain a high level of noise. Other results also exhibited noisy profiles, but each was entirely different from the others. Therefore, the doping is effective for the induction of higher current. However, the IPMC should be well dried, otherwise, the inducced current becomes uncontrollable. These observations further suggest that the bending behavior of wet BP-IPMC is difficult to control. Therefore, wet-state IPMC is considered unsuitable for the purposes of our study. In fact, we previously reported that the bending controllability of IPMC improves in the dry state, but even the slight humidity increase deteriorates such a preferable characteritic as mentioned at the end of Section 3.2.2 [37], and the BP-IPMC characteritics are also affected by the environmental humidity. The study on bending controllability is our next task, and we are currently working on it. We would like to present some preliminary results on this topic in Appendix B. A more detailed discussion will be provided in our next paper.

## 4. Conclusions

Past investigations on IPMC bending suggest that its characteristics are largely governed by the current (or charge) applied to it. In other words, the greater the imposed current (or charge), the larger the bending curvature becomes. Based on this idea, we investigated ways to enhance current induction within the IPMC body in order to achieve effective bending. Our findings indicate that the following factors are important for promoting effective current induction in IPMC: (i) electrically tight contact between the electrode metal and the ion-exchange membrane surface, (ii) doping the IPMC body with the appropriate mobile ions, and (iii) supplying external oxygen to the IPMC body. We experimentally confirmed that effective current induction indeed resulted in the significant bending of our IPMC, which was made of an amphoteric ion-exchange membrane coated with silver. However, we also found that current induction in IPMC is highly sensitive to its degree of wetness and can even be influenced by ambient humidity. Therefore, precise control of IPMC bending through current (or charge) regulation requires simultaneous control of environmental conditions. Although controlling environmental conditions is not an easy task, it constitutes our next challenge.

## Figures and Tables

**Figure A1:**
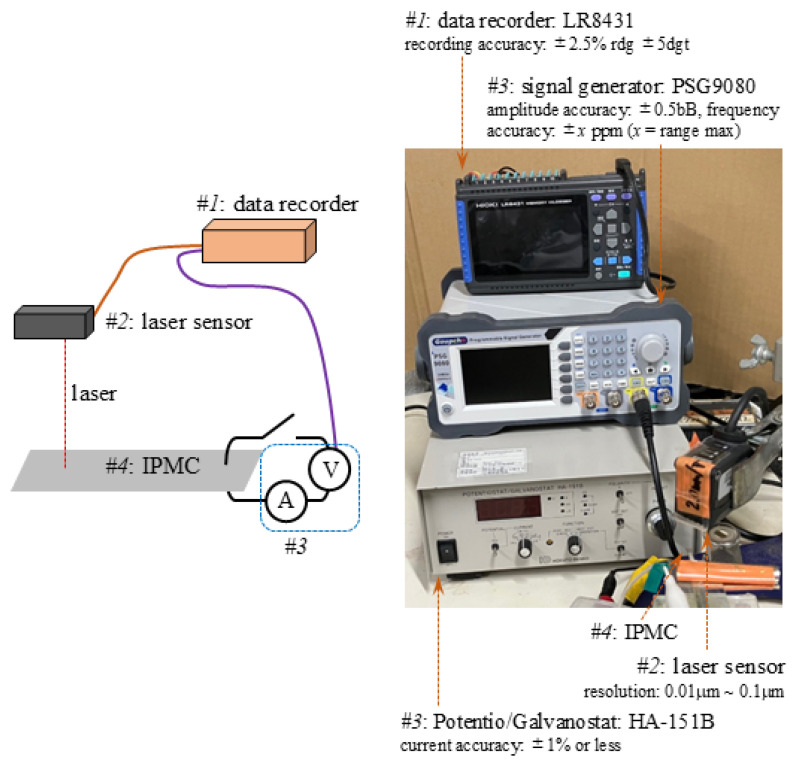
Illustration of the experimental setup (Figure 2) and its correspondence to the actual experimental components. ♯1: Data recorder (LR8431; HIOKI E.E. Corporation, Japan; recording accuracy: ±2.5% rdg ±5 dgt), ♯2: Laser sensor (IL-030; Keyence, Japan; resolution: 0.01 μm–0.1 μm), ♯3: Devices for voltage and current control and measurement, including a potentiostat/galvanostat (HA-151B; Meiden Hokuto Corporation, Japan; current accuracy: ±1% or less) and a signal generator (PSG9080; Hangzhou Junce Instruments Co., Ltd., China; amplitude accuracy: ±0.5 dB, frequency accuracy: ±*x* ppm (*x* = range max)), and ♯4: IPMC specimen clamped by the fixture.

**Figure A2:**
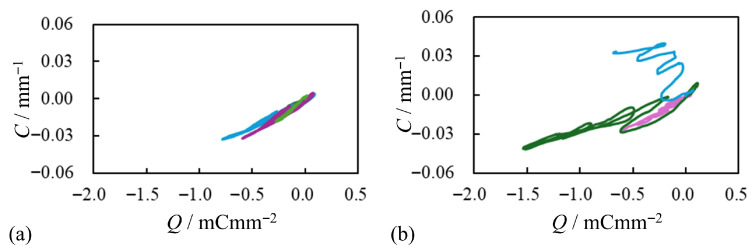
Curvature-Charge relatiopnship of BP-IPMC obtained by conducting the the same experiemnt described in the Section 3.1. (**a**) BP-IPMC in the well-dried state. (**b**) BP-IPMC in the wet state.

## Data Availability

The raw data supporting the conclusions of this article will be made available by the authors on request.

## Appendix B. Stability of Bending of BP-IPMC in the Dry and Wet State

For the purpose of verifying the bending stability of BP-IPMC, we repeated the same experiment and analysis as in Section 3.1. Figure A2a shows the four data sets, but all of them nearly overlap on the same line, with one data set being nearly invisible because it is covered by the other three curves. This indicates that the bending of BP-IPMC in the well-dried state exhibits quite stable and reproducible bending characteristics. On the other hand, the bending of BP-IPMC in the wet state becomes unpredictable, as shown in Figure A2b. Figure A2b presents three data curves, one of which deviates significantly from the other two. This suggests that the wetting treatment on the BP-IPMC deteriorates its bending controllability. Further research outcomes on bending stability will be reported in the next paper we plan to publish.
